# Fabrication, Characterization, and Cytotoxicity of Thermoplastic Polyurethane/Poly(lactic acid) Material Using Human Adipose Derived Mesenchymal Stromal Stem Cells (hASCs)

**DOI:** 10.3390/polym10101073

**Published:** 2018-09-28

**Authors:** Anna Lis-Bartos, Agnieszka Smieszek, Kinga Frańczyk, Krzysztof Marycz

**Affiliations:** 1 AGH University of Science and Technology, Department of Biomaterials and Composites, Faculty of Materials Science and Science and Ceramics, Krakow 30-059, Poland; annal@agh.edu.pl; 2 Department of Experimental Biology, Wroclaw University of Environmental and Life Sciences, Wroclaw 50-375, Poland; agnieszka.smieszek@upwr.edu.pl; 3 AGH University of Science and Technology, Faculty of Electrical Engineering, Automatics, Computer Science and Biomedical Engineering, Krakow 30-059, Poland; kinga.franczyk@poczta.onet.pl; 4 Faculty of Veterinary Medicine, Equine Clinic-Equine Surgery, Justus-Liebig-University, Gießen 35392, Germany

**Keywords:** thermoplastic polyurethane, poly(lactic acid), polymer blends, stem cells, osteochondral tissue engineering

## Abstract

Thermoplastic polyurethane (TPU) and poly(lactic acid) are types of biocompatible and degradable synthetic polymers required for biomedical applications. Physically blended (TPU+PLA) tissue engineering matrices were produced via solvent casting technique. The following types of polymer blend were prepared: (TPU+PLA) 7:3, (TPU+PLA) 6:4, (TPU+PLA) 4:6, and (TPU+PLA) 3:7. Various methods were employed to characterize the properties of these polymers: surface properties such as morphology (scanning electron microscopy), wettability (goniometry), and roughness (profilometric analysis). Analyses of hydrophilic and hydrophobic properties, thermogravimetric analysis (TGA), and differential scanning calorimetry (DSC) of the obtained polymer blends were conducted. Tensile tests demonstrated that the blends exhibited a wide range of mechanical properties. Cytotoxicity of polymers was tested using human multipotent stromal cells derived from adipose tissue (hASC). In vitro assays revealed that (TPU+PLA) 3:7 matrices were the most cytocompatible biomaterials. Cells cultured on (TPU+PLA) 3:7 had proper morphology, growth pattern, and were distinguished by increased proliferative and metabolic activity. Additionally, it appeared that (TPU+PLA) 3:7 biomaterials showed antiapoptotic properties. hASC cultured on these matrices had reduced expression of Bax-α and increased expression of Bcl-2. This study demonstrated the feasibility of producing a biocompatible scaffold form based on (TPU+PLA) blends that have potential to be applied in tissue engineering.

## 1. Introduction

Tissue engineering (TE) and material science are valuable tool in regenerative medicine [1,2]. Until now, tissue engineering has been successfully used to obtain promising materials dedicated to the regeneration of various tissue, including soft and hard tissues [3,4]. Particular attention is paid to the development of polymer-based matrices, especially in terms of tailoring their chemical, physical, and biological properties, in order to obtain the most cytocompatible products [5,6,7,8]. Moreover, polymers are important and attractive biomaterials due to high application potential, including scaffold design using three dimensional (3D) technology, which is fundamental in personalized medicine [9,10]. Obviously polymers used in the field of tissue engineering should be characterized in terms of their biocompatibility, biodegradability, but also immunomodulatory and biomechanical properties [11,12]. Both poly(lactic acid) (PLA) and thermoplastic polyurethane (TPU) are synthetic polymers commonly used in biomedical applications, however they differ in mechanical properties [5,13,14]. The former polymer has rigid mechanical properties, while the latter is characterized by flexible mechanical properties [6]. It is worth mentioning that appropriate mechanical properties of porous scaffolds determine various important aspects of tissue healing such as augmentation and filling of the defect, further promoting proper tissue repair [15]. Thus, the current TE perspectives are focused on manufacturing an ideal scaffold, characterized by mechanical features that would perfectly match the native tissue to be repaired [16].

Poly(lactic acid) (PLA)-based scaffolds have been widely used to promote bone and cartilage regeneration as well as to provide a substitute for blood vessels [7]. However, PLA scaffolds are characterized by brittle properties and relative high strength, which limits their applicability [5,17]. In turn, thermoplastic polyurethane (TPU) has been characterized as a polymer with a wide suitability in medical applications, not limited to the promotion of bone and cartilage regeneration [5,6,8]. For example TPU can be used to repair and reconstruct mechanically active soft tissues including blood vessels, cardiac walls and valves, or urinary bladders and skin [5]. TPU scaffolds are also characterized by high elongation, an attractive Young’s modulus, as well as excellent abrasion and tear resistance. Therefore, it seems reasonable to combine thermoplastic polyurethane with poly(lactic acid) (TPU+PLA) to create an attractive hybrid material that could have a wider spectrum of applicability in the field of regenerative medicine [5,8]. We decided to test TPU+PLA blend cytocompatibility using the model of human multipotent stromal cells originated from adipose tissue (hASCs). This aspect is important bearing in mind that current efforts in TE are also aimed on adapting TPU+PLA blend to obtain biomaterials suitable for multiple tissue applications. The multipotent character of those cells refers to their ability to differentiate, inter alia into bone and cartilage-forming cells [18,19,20].

Recently, adipose derived multipotent stromal cells have increasingly been used for orthopedic-based tissue engineering [21,22,23]. ASCs are for various reasons recognized as a powerful therapeutic tool in the field of regenerative medicine. ASCs are characterized by high proliferative and secretory activity [24,25]. The fact that ASCs are able to differentiate into osteoblasts, chondroblasts, and adipocytes, makes them very attractive in terms of broad range of potential clinical application [18,20]. More importantly, they exhibit an immunomodulatory ability, improving the regenerative capacity during wound healing [24]. Bearing in mind all aforementioned facts, hASCs are also commonly applied as a model for testing of biomaterials cytocompatibility [26,27,28]. It has been previously shown by our and other groups that cellular plasticity of hASCs is a feature that enables to perform valuable screening tests of various biomaterials, both natural and synthetic polymers, as well as metal-based implants [8,26,27,28,29,30]. The analysis of cytophysiological properties of hASCs such as morphology, proliferation, and metabolic activity in cultures with newly designed scaffolds is particularly important when potential autologous transplants are considered [31,32].

This study was aimed at the fabrication and characterization of (TPU+PLA) blends prepared by dissolving them in mixed organic solvents. The obtained (TPU+PLA) blends were used to produce films characterized by different Young’s moduli (*E*) in the MPa range and various chemical compositions. Generally, the following types of polymer blends were prepared (TPU+PLA) 7:3, (TPU+PLA) 6:4, (TPU+PLA) 4:6, and (TPU+PLA) 3:7. We decided to investigate biomaterials’ morphology, contact angle, and mechanical properties because surface and volume wettability of scaffolds intensively affects cell attachment and viability. We also decided to test the cytotoxicity of the obtained biomaterials using hASC model due to the fact that our goal was to obtain scaffolds for progenitor cells and matrices that could be potentially used in multiple tissue applications. Moreover, the proliferation of hASCs was studied as an effect of surface stiffness, which depends on the polymer blend composition.

## 2. Materials and Methods

Thermoplastic polyurethane—TPU (Ellastolan 1102A, PAN, Wroclaw, Poland) is a high flexible elastomers that provides elasticity to the designed blends. Its glass transition temperature (*T*_g_) is −50 °C (temperature founded by DSC test). Poly(lactic acid)—PLA (PURASORB, Nederland) was chosen to improve the rigidity (physical stability) of the polymer blends. Its glass transition temperature is 60 to 63 °C (based on completed DSC test). The *N*’*N* dimethylformamide (DMF) and dichloromethane (DCM) of analytical grade, as solvents were purchased from Sigma-Aldrich (Poland, Poznan).

### 2.1. Polymer Blends Fabrication

Polymer films (cell culture matrices) were fabricated using a solvent casting method. Five percent thermoplastic polyurethane/poly(lactic acid) combinations were prepared by dissolving TPU and PLA in *N*’*N* dimethyloformamide and dichloromethane mixture. Processing conditions: (TPU+PLA) different composition (5% (*w*/*w*)) were dissolved in an 8:2 mixture of DMF and DCM by warming at ca. 35 °C with magnetic stirring until the polymer solution became clear. This temperature was above the boiling point of DCM (39.6 °C) and glass transition temperature of poly(lactic acid) (~60 °C). Polyurethane and poly(lactic acid) were selected because of its known biocompatibility, easy of processing, and extensive use in the previous studies [26]. The polymers solution was sealed in glass bottle prior to use and used immediately after opening to ensure that minimal solvents were lost via evaporation. Polymer films were prepared by solvent casting onto glass Petri dish as previously described [26]. The following types of polymer blend were prepared (TPU+PLA) 7:3, (TPU+PLA) 6:4, (TPU+PLA) 4:6, and (TPU+PLA) 3:7. In comparison, (TPU+PLA) films were also fabricated by this technique but only DMF (with a high boiling point: 153 °C) was used as a solvent with low evaporation rate.

### 2.2. Biomaterials Characterization

#### 2.2.1. Scanning Electron Microscopy (SEM)

The surface morphology and microstructure of the prepared polymer films was observed by scanning electron microscopy (SEM). SEM imaging was performed using Nova Nano SEM 200 microscope (FEI Europe Company, Hillsboro, OR, USA). The samples were fractured by a surgical blade. Prior to imaging scaffolds were made conductive with a thin layer of carbon by using a sputter-coater. Scanning electron microscopy was used for studying the surface morphology of determined by polymer blend composition.

#### 2.2.2. Surface Wettability

Goniometry was used to measure the surface contact angles of the various polymer blend formulations. This method provides information on surface hydrophilicity or hydrophilicity; important factors for in vitro tests. The surface wettability of prepared thin polymer films was measured by water contact angle analysis. Contact angle of the (TPU+PLA) films were measured on air atmosphere and at room temperature. The water contact angle of thin polymer samples was measured via sessile drop technique using a goniometer DSA 10 Mk2 (Kruss, Hamburg, Germany) equipped with internal image analysis software. Distilled water (purchase from Sigma Aldrich, Poznan, Poland) drops of the volume of 0.5 μL were put on each dry polymer films and the static contact angle was measured immediately. Contact angle was calculated by averaging the results of ten measurements (ten region in specimen). Wetting process was recorded using a digital camera.

#### 2.2.3. Surface Roughness

Topography (surface roughness, as important feature for cell culture) of obtained polymer films were characterized by profilometry (Hommelwerke profilometer, Villingen-Schwenningen, Germany). The average roughness (*R*_a_) of polymer samples were determined. The various regions of each materials were examined. The presented results herein are the average of ten measurements (±standard deviation).

#### 2.2.4. Mechanical Properties Evaluation

The mechanical properties of the (TPU+PLA) different blends were determined by tensile test. Mechanical properties of polymer thin films were determined in tensile uniaxial mode with the use ZWICK universal testing machine model 1435, using the crosshead speed of 5 mm/min. Samples examined had the dimensions 60 mm × 5 mm. Depending on the polymer blend composition the thickness of the films varied from 50 μm to 0.1 mm. Young’s modulus (*E*) of the polymer blends was determined as the slope of the straight line stress–strain curve. Young’s modulus were obtained as the mean and standard deviation of ten measurements.

#### 2.2.5. Thermal Degradation Test

The thermal properties of obtained polymer blends and pure polymers were examined by thermogravimetric analysis (TGA) and differential scanning calorimetry (DSC) The thermal degradation (thermostability) studies of the PLA, TPU, and (TPU+PLA) blends were carried out in NETZSCH analyzer, as well as the thermal properties of the blends measured by DSC. The samples (10 mg) were degraded under nitrogen atmosphere at the heating rate of 10 °C/min. The samples were scanned from 20 to 600 °C for TGA and DSC curve definition.

#### 2.2.6. Sterilization

Pure polymer and (TPU+PLA) blend films were cut into small disks (15 mm in diameter) with the aid of leather borer in order to locate the disks into 24 well tissue culture plates. All the samples were prewetted and sterilized in ethanol for 10 min, and then ethanol was evaporated. Dried disk was packed and sterilized by peroxide hydrogen cold plasma sterilization method.

### 2.3. Assessment of Biomaterials Cytocompatibility Using Model of Human Adipose Derived Mesenchymal Stromal Stem Cells (hASCs)

#### 2.3.1. The Characteristics of Human Adipose-derived Stromal Cells (hASCs) Used for the Experiment

The cells used for the current study were isolated using well-established method described previously in detail [26,33]. Subcutaneous adipose tissue was collected both from male and female subjects, age range 20 to 33 (*n* = 8, mean age 24 ± 1.5 years). All donors had given written consent prior to the procedure. The characteristic phenotype indicating on mesenchymal origin of hASCs was also determined using well established protocol and accordingly to the criteria established by International Society of Cellular Therapy [31,34]. The immunophenotyping was performed after third passage (p3) using protocol published previously [27]. The cells used for the experiment expressed the specific surface markers such as CD44, CD73, CD90, and CD105. Moreover hASCs used here did not expressed markers of hematopoietic origin i.e., CD34 and CD45. The cells underwent the osteogenic, chondrogenic, as well as adipogenic differentiation exhibiting their multipotent character.

#### 2.3.2. The Metabolic Activity and Proliferation of hASCs Cultures on Biomaterials

The cytocompatibility assay was performed in 24-well dishes covered with TPU+PLA films. For the test cells were inoculated at density equal 45,000 cells per well, suspended in a 0.5 mL of complete growth medium (CGM) consisting of Dulbecco’s Modified Eagle’s Medium with nutrient F-12 Ham supplemented with 10% of fetal bovine serum (FBS) and 1% antibiotic-antimycotic solution. Each culture was prepared at least in nine replicates. Cultures were propagated in aseptic conditions in CO_2_ incubator (37 °C, 95% humidity and 5% CO_2_). The metabolic activity of hASCs was measured 120 h of cultures using resazurin-based assay (Alamar Blue dye, Sigma Aldrich, Poznan, Poland). The test was conducted accordingly to the protocol described in previously and in accordance with the manufacturer’s suggestions [26]. The metabolic activity of hASCs cultured on biomaterials was compared with the metabolic activity of cultures propagated on polystyrene (P/S) and expressed as metabolic factor (MF). The proliferation of cells was measured by detection of 5-bromo-2-deoxyuridine (BrdU) incorporation test. For this purpose after 120 h of cultures the cells were detached from the biomaterials surface and polystyrene (P/S) using TrypLE Express solution. Cells were then washed with Hanks’s Balanced Salt Solution (HBSS) containing 2% FBS, centrifuged, and resuspended at the total amount in a 96-well plates in six replications. Next, plates were cultured for 24 h and additionally treated with BrdU reagent and incubated overnight at 37 °C in humidified atmosphere of 5% CO_2_ and 95% air. The BrdU assay was then performed using the general protocol, described in detail previously [35]. The reagents used for cell cultures and metabolic assay derived from Sigma Aldrich, Poznan, Poland. The BrdU assay as well as TrypLE Express solution were purchased from Abcam (Cambridge, UK) and Thermo Fisher Scientific (Waltham, MA, USA), respectively.

#### 2.3.3. The Morphology of hASCs Cultures on Biomaterials

The attachment of hASCs onto biomaterials surface, as well as the morphology and growth pattern of cultures were determined after 120 h of cells propagation. The microscopic evaluation of hASCs on biomaterials surface was performed using scanning electron microscope (SEM, Auriga 60, Zeiss, Oberkochen, Germany) and confocal microscope Cell Observer SD (Zeiss, Oberkochen, Germany) as described previously [36,37,38]. To prepare samples for observations the cultures were fixed with 4% paraformaldehyde. The specimens were either dehydrated in graded ethanol series (concentrations ranges from 50% to 100%, every 10%) for SEM analysis or stained with fluorescent dyes for confocal microscope observations. To visualize the nuclei and cytoskeleton the cultures were stained with diamidino-2-phenylindole (DAPI; 1:800) and atto-488-labeled phalloidin (1:800). The staining was performed using well established protocols [8,26,30]. All reagents used during this protocol derived from Sigma Aldrich, Poznan, Poland.

#### 2.3.4. The Analysis of Antiapoptotic and Proapoptotic Bcl-2 Proteins

After 120 h of culture the cells were harvested from the biomaterials and treated with TRI Reagent (Sigma Aldrich, Poznan, Poland). The total RNA isolation was performed using well-established protocol by Chomczynski and Sacchi [39]. Obtained RNA was diluted in DEPC-treated water, and its quantity and purity was determined using NanoDrop 8000 (ThermoFisher Scientific, Waltham, MA, USA). The genomic DNA (gDNA) traces were removed using prepared using PrecisionTMDNase kit (Primerdesign Ltd., Cambridge, UK). The cDNA was obtained from 0.5 µg of RNA using transcription Tetro cDNA Synthesis Kit (Bioline Reagents Ltd., London, UK). For qPCR the total volume of reaction mix (SensiFast SYBR & Fluorescein Kit; Bioline Reagents Ltd., London, UK) was 20 μL. The cDNA did not exceed 10% of the final PCR reaction volume. The specific primers used in concentration equal 400 nM derived from Sigma Aldrich (Poznan, Poland).

The expression of B-cell lymphoma 2 (Bcl-2) was detected using the following primers forward 5′-ATCGCCCTGTGGATGACTGAG-3 and reverse 5′-CAGCCAGGAGAAATCAAACAGAGG-3′ (129 bp). The sequences of the primers used for Bcl-2-associated X protein (Bax-α) amplification were as follows forward 5′-ACCAAGAAGCTGAGCGAGTGTC-3′ and reverse 5′-ACAAAGATGGTCACGGTCTGCC-3′ (365 bp). The relative gene expression analysis (normalized quantity/Qn) was calculated in relation to a housekeeping gene, i.e., glyceraldehyde 3-phosphate dehydrogenase. The following primers were used forward 5′-GTCAGTGGTGGACCTGACCT-3′, reverse 5′-CACCACCCTGTTGCTGTAGC-3′ (256 bp). The qPCR was performed using CFX Connect Real-Time PCR Detection System (Bio-Rad Polska Sp. z.o.o., Warszawa, Poland) using protocol precisely described elsewhere [40,41]. For the total Bax-α measurement cells were detached from biomaterials and lysed using ice-cold RIPA extraction buffer with the cocktail of protease and phosphatase inhibitors. The total protein in cell lysates was quantified using the Bicinchoninic Acid Kit Assay Kit (Sigma Aldrich, Poznan, Poland). The concentration of Bax-α was determined using sandwich ELISA derived from R&D Systems (Minneapolis, MN, USA). For each measurement samples with concentration of 30 μg/mL of total protein were used. The protocol of protein assays were conducted on abstract-treated cells according to the manufacturer’s instructions. The absorbance was read on a microplate reader Spectra MaxPlus (Molecular Devices, Syngen, Wroclaw, Poland) at 450 nm with wavelength correction set to 540 nm. All tested samples as well standards were measured in three replicates.

### 2.4. Statistical Analysis

The results obtained from biological assays are expressed as the mean values with standard deviation (SD). One-way analysis of variance and Dunnett’s post hoc analysis was done to determine statistical significance (*p* < 0.05). The data were analyzed using GraphPad Software (Prism 5.04, GraphPad Software, La Jolla, CA, USA).

## 3. Results

### 3.1. Fabrication and Microstructure

Polyurethanes are widely existing synthetic biodegradable or nondegradable hydrophilic polymers. It can be used to composed with more hydrophilic poly(lactic acid) polymer blends to improve biocompatibility, degradability, hydrophilicity, and cell affinity of polymeric blend materials. A simple technique to fabricate a various composition of blends was solvent casting method. However, polyurethanes are generally insoluble in common organic solvent. Dichloromethane is usually used to fabricate PLA films and other forms of scaffolds for its good solubility with PLA family polymers and its fast evaporation rate. Some polyurethanes are soluble in N’N dimethyloformamide with a high boiling point (*T*_B_ = 153 °C). For preparation of different form of (TPU+PLA) blends we use DMF mixed with a DCM with a lowest boiling point (less than 40°). In this study, a mixed solvent of DCM and DMF (*v*/*v* = 8:2) was tested as a suitable solvent for both PLA and TPU. It has been found that TPU are soluble in organic solvent mixtures when the concentration of DCM is 20% (*v*/*v*). Moreover the DMF and DCM mixture can run the speed of solvent evaporation from polymer based materials. Subsequently, the more energetic evaporation of DCM might result in a better porous structure. The microstructure of the polymer blends and control samples was observed by a scanning electron microscopy. Figures in Table 1 show examples of the SEM images of polymer films. A good integration of polyurethane and poly(lactic acid) phase can be found in the SEM magnification. Microscopy examination of the polymer blend specimens from all four compositions confirm their surface roughness.

### 3.2. Surface Wettability

The surface hydrophilic or hydrophobic of polymer thin films matrices has a major influence on cell adhesion and proliferation behavior. The hydrophilicity of matrices has an influence on the surface energy, which might influence the serum proteins to adhere on the matrices, and in turn govern the biological response, such as cell adhesion, proliferations, and differentiations. Table 2 shows result of contact angle measurement for pure TPU, PLA, and (TPU+PLA) films. Generally the hydrophilicity of PLA films was higher than TPU and its blends, proving that the hydrophilicity of polymer blends increased with increasing poly(lactic acid) concentration. The wettability of (TPU+PLA) polymer films was found from 80° for PLA films to 123° for TPU films. The significant differences in the contact angle values of TPU, PLA, and (TPU+PLA) blends were observed. Results of our studies show that the incorporation of PLA in TPU has major effect towards the hydrophilicity of (TPU+PLA) films, compared with the hydrophobic TPU films.

### 3.3. Surface Roughness

Topography, among other chemical and physical factors such as substrate stiffness and polymer blend composition, is known to have a great influence on cell behavior. Optimization of topographical features, such as surface roughness, is the one of the key strategy to obtain the best cellular scaffolds for various tissue applications. This work investigated the influence of polymer blend composition on surface roughness. The average roughness (*R*_a_) of the polyurethane, poly(lactic acid), and (TPU+PLA) blends are presented in Table 2. The average roughness of all polymer blends was from 86 μm for (TPU+PLA) 7:3 to 102 μm for (TPU+PLA) 3:7. As a rule, for the blends with higher concentration of PLA surface roughness is higher. Polymer blends with higher addition of elastic TPU are more flattered surface, as well as pure polyurethane films.

### 3.4. Thermal Properties of (TPU+PLA) Blends

The TGA and DSC curves presented in Supplementary Figure S1 show the thermal degradation behavior of each the (TPU+PLA) blends and control samples heated to 600 °C under a nitrogen atmosphere. TGA curves show similar profiles, as well as DSC curves, with thermal decomposition opening at 200 °C and extending to 450 °C. The maximum thermal decomposition of polymer samples was observed from 300 to 400 °C (all samples lost 50 percent of mass).

### 3.5. Tensile Test

At this point, we test the elastic moduli, which plays a crucial role in cell culture. The substrate elastic moduli might regulate the adhesion, morphology, proliferation, or differentiation of cells. Four kinds of (TPU+PLA) blend films and control material samples were subjected to the measurement of tensile tests, and Young moduli examination results are presented in Table 3. The strain–stress behavior of (TPU+PLA) films shows that PLA with TPU caused a reduction in mechanical stress and elasticity. The percent value of elongation of (TPU+PLA) films were higher compared to pure PLA indicating a good deformability and flexibility of obtained polymer blends. However, the percent elongation of (TPU+PLA) blends decreased significantly due higher PLA concentration in (TPU+PLA) blended polymeric films. Pure PLA films had a high tensile modulus (3.44 GPa) and exhibited an extremely low-elongation-at-break due to their inelastic nature. TPU film samples did not break during the tensile test even when the strain reached 500%.

In summary, the ratios of 7:3 and 6:4 of (TPU+PLA) blend were therefore specifically chosen for the preparation of elastic scaffolds for cartilage zone and chondrocyte cell culture, with 3:7 and 4:6 of (TPU+PLA) chosen for more rigid scaffolds, for subchondralbone scaffolds, and osteoblast cell culture. All (TPU+PLA) compositions were selected for stem cell culture. Scaffolds have varying properties in different regions of the scaffolds for optimizing ingrowth of different tissues and cells. It is known that various topography, elasticity, and chemistry are suitable for ingrowth of different tissues. We carried out material characteristics and in vitro experiments evaluating the capability of (TPU+PLA) blends to modulate the behavior of stem cells for osteochondral tissue engineering.

### 3.6. The In Vitro Evaluation of Cytotoxicity of Obtained Biomaterial

#### 3.6.1. Proliferation and Metabolic Activity of hASCs in Cultures with Obtained Biomaterials

**Figure 1:**
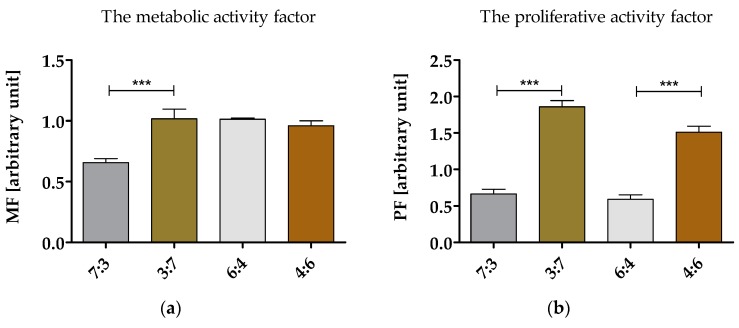
Metabolic and proliferative activity of hASCs cultivated onto investigated (TPU+PLA) films. The metabolic and proliferative factor was determined in relation to the cultures propagated on polystyrene. The analysis was performed after 120 h of hASCs culture in obtained matrices. An asterisk marks a statistically significant difference (*** *p* < 0.001).

Both metabolic and proliferative activity were determined after 120 h of cell propagation in cultures with biomaterials (Figure 1a,b, respectively). The analysis of hASCs metabolism in cultures with (TPU+PLA) blends revealed that only (TPU+PLA) in ratio 7:3 may exert the cytotoxic effect toward hASCs lowering significantly the metabolic activity of the cells. Furthermore, the analysis of the proliferation status of hASCs showed that both (TPU+PLA) 7:3 and (TPU+PLA) 6:4 may inhibit the proliferative properties of hASCs. Bearing in mind both metabolic and proliferative potential of hASCs in cultures with (TPU+PLA) blends, the most cytocompatible matrices were characterized by lowered content of TPU, i.e., (TPU+PLA) 3:7 as well as (TPU+PLA) 4:6. Obtained results are in good agreement with the study of Mi et al. who developed the TPU/PLA scaffolds fabricated by microcellular injection molding. In this study cultures of 3T3 fibroblast cell line exhibited slightly higher cell viabilities in the scaffolds with more PLA content [6]. The increased proliferative activity might be associated with the lowest water contact angle noted for (TPU+PLA) 3:7 as well as for (TPU+PLA) 4:6 films, which was equal to 87.7 ± 1.8 and 86.9 ± 2.6, respectively. The obtained data correlates with our previous research and that of others, where the increased proliferative activity of stromal stem cells was related to water contact angle [8]. Moreover, the highest metabolic activity, that was measured on the 5th day of experimental culture, was found in hASCs cultured onto (TPU+PLA) 3:7, (TPU+PLA) 6:4, (TPU+PLA) 4:6, and (TPU+PLA) 4:6. The results presented here support previous findings that polymer bland matrices (TPU+PLA) are not toxic for hASCs and may serve as a scaffolds for progenitor cells [8,29]. This may shed a promising light for the further future application of (TPU+PLA) material in a wide range biomedical applications, both for hard and soft tissue healing [7].

#### 3.6.2. The Morphology and Growth Pattern of hASCs Cultured on Obtained (TPU+PLA) Films

**Figure 2:**
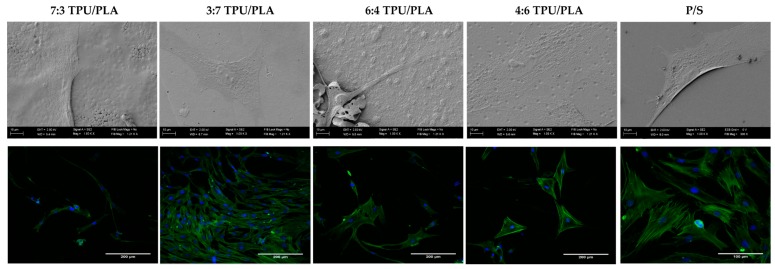
The morphology of hASCs cultured onto obtained (TPU+PLA) blends. The morphology of cells was visualized using scanning electron microscope (SEM) and confocal microscope. The SEM images (revealed the adhesion rate of hASCs on tested (TPU+PLA) blends, and some biomimetic features of films). For confocal observations, cells were stained with DAPI, to localize the nuclei (blue dots) and with phalloidin atto-488 (green, actin cytoskeleton). SEM microphotographs were captured under 1000× magnification (scale bar is 10 μm), while confocal observations were performed at magnification 40× (scale bar 200 µm).

The analysis of hASCs morphology and growth pattern in cultures with TPU+PLA films clearly indicated on the most cytocompatible blend (Figure 2). The proper morphology of hASCs, as well as growth pattern, was noted in culture with (TPU+PLA) 3:7 matrices. The cells in this culture were spindle-shape or multipolar, which is typical morphotype for multipotent stromal cells of mesenchymal origin. The analysis of growth pattern performed using confocal microscope revealed that TPU+PLA) 3:7 matrices promoted cell-cell interactions. The hASC cultured on (TPU+PLA) 3:7 formed dense active layer of closely arranged cells. These cultures were characterized by cells with well-developed cytoskeleton and centrally located nuclei. Moreover hASCs, were distributed evenly on the (TPU+PLA) 3:7 matrices. The increased confluence of hASCs on (TPU+PLA) 3:7 matrices correlates with their high proliferative activity noted in cultures on those biomaterials. The morphology of cells propagated on remaining (TPU+PLA) films, i.e., (TPU+PLA) 7:3, (TPU+PLA) 6:4, and (TPU+PLA) 4:6 was typical for hASCs, and fibroblast-like cells were noted, despite that the cells in these cultures did not reveal tendency for the formation of intracellular connections. Obtained data stands in good agreement with previous findings, showing that (TPU+PLA) blends with the higher content of PLA positively affects the adhesion and morphology of progenitor cells, both of adipose origin as well as derived from olfactory bulb [8,29,42].

#### 3.6.3. Evaluation of Bax/Bcl-2 Ratio and Total Bax-α in hASCs Cultured on (TPU+PLA) Blends

**Figure 3:**
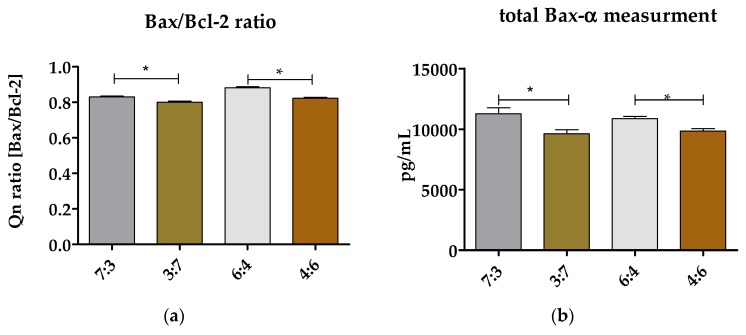
The analysis of Bax/Bcl-2 ratio (**a**) and total concentration of Bax-α (**b**). The transcripts levels were determined using RT-qPCR method, while total Bax-α was evaluated using ELISA technique. An asterisk was used to mark a statistically significant differences determined between tested groups (* *p* < 0.05).

The analysis of the transcript levels indicated that the higher content of PLA may influence on increased mRNA level of antiapoptotic Bcl2 gene (Figure 3a). The PLA is described as less cytotoxic polymer than TPU, thus adequate content of TPU and PLA may improve biological activity of blend [6,7,13]. The high Bax/Bcl-2 ratio was noted in hASCs cultures propagated onto (TPU+PLA) 7:3 and (TPU+PLA) 6:4. The increase mRNA level of Bax-α in those cultures reflected also on the accumulation of Bax-α protein in cells, which was determined using the ELISA technique (Figure 3b). The high expression of proapoptotic Bax-α in cultures on biomaterials with the dominant content of TPU correlates with the results of proliferative activity of hASCs. Obtained data clearly indicate, that the proper (TPU+PLA) ratio may exert pro-proliferative and antiapoptotic effect toward multipotent progenitor cells derived from adipose tissue.

## 4. Conclusions

In this study we developed blend (TPU+PLA) films with a weight ratio of 7:3, 6:4, 4:6, and 3:7. The results reported in this study demonstrated that the concentration of PLA in the polymer blends was strongly affected by the properties of polymer matrices. Mechanical tests confirmed the very high tensile range of the (TPU+PLA) scaffolds fabricated by the solvent casting method, which can potentially be used in osteochondral tissue engineering in many tissue applications. An in vitro proliferative and metabolic assay using human adipose-derived mesenchymal stem cells (hASCs) showed that (TPU+PLA) 3:7 was the most cytocompatible. Moderate cytocompatibility was also observed for (TPU+PLA) 4:6 matrices. Both (TPU+PLA) 3:7 and (TPU+PLA) 4:6 can also improve hASCs viability by increasing the expression of antiapoptotic Bcl-2 and decreasing proapoptotic Bax-α, both at the mRNA and protein level. The obtained data clearly indicate, that our materials could be used as sufficient scaffolds for progenitor cells like hASCs. The different ratio of TPU to PLA can significantly modulate proliferative and metabolic activity, adhesion as well as growth pattern and cell-cell contacts. Further research is required to test whether our concept of using different ratio of (TPU+PLA) films in different physical forms, i.e., sponges or fibrous matrices, might differentially induce osteogenic and/or chondrogenic differentiation.

## Figures and Tables

**Table 1:** SEM images of thin films made from PLA, TPU and (TPU+PLA) blends.

**TPU 100% (control)**	**(TPU+PLA) 7:3**	**(TPU+PLA) 6:4**
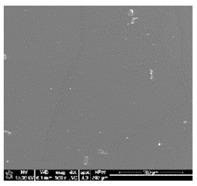	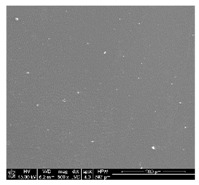	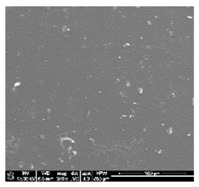
**(TPU+PLA) 4:6**	**(TPU+PLA) 3:7**	**PLA 100% (control)**
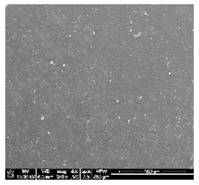	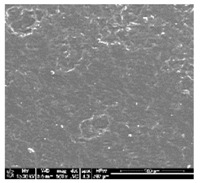	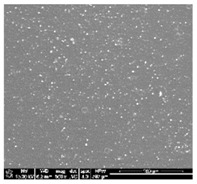

**Table 2:** The surface properties of polyurethane, poly(lactic acid), and their blends.

**TPU 100% (control)**	**(TPU+PLA) 7:3**	**(TPU+PLA) 6:4**
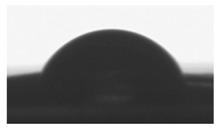	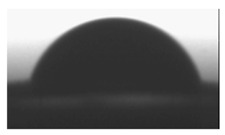	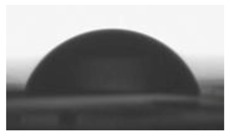
*θ* = 123.2 ± 1.8 [°]	*θ* = 118.9 ± 2.3 [°]	*θ* = 96.3 ± 1.3 [°]
*R*_a_ = 72.1 ± 1.2 [µm]	*R*_a_ = 86.1 ± 1.7 [µm]	*R*_a_ = 83.1 ± 2.1 [µm]
**(TPU+PLA) 4:6**	**(TPU+PLA) 3:7**	**PLA 100% (control)**
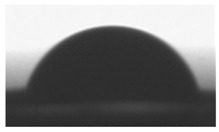	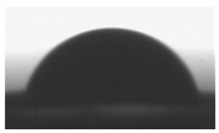	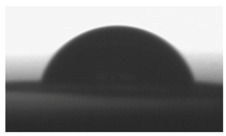
*θ* = 87.7 ± 1.8 [°]	*θ* = 86.9 ± 2.6 [°]	*θ* = 79.9 ± 1.1 [°]
*R*_a_ = 97.4 ± 1.9 [µm]	*R*_a_ = 102.5 ± 1.6 [µm]	*R*_a_ = 115.8 ± 2.3 [µm]

**Table 3:** Tensile Young’s moduli of thin films.

Polymer Composition	Tensile Young’s Modulus for Thin Films [MPa]
**TPU 100% (control)**	29.6 ± 0.6
**(TPU+PLA) 7:3**	84.5 ± 0.5
**(TPU+PLA) 6:4**	162.4 ± 1.1
**(TPU+PLA) 4:6**	215.6 ± 1.9
**(TPU+PLA) 3:7**	237.0 ± 3.2
**PLA 100% (control)**	3.44 ± 2.5 [GPa]

## Supplementary Materials

The following are available online at http://www.mdpi.com/2073-4360/10/10/1073/s1, Figure S1: TGA and DSC curves of different (TPU+PLA) blends: 7:3, 6:4, 4:6 and 3:7.
