# Supplementary figures and images for: Fabrication, Characterization, and Cytotoxicity of Thermoplastic Polyurethane/Poly(lactic acid) Material Using Human Adipose Derived Mesenchymal Stromal Stem Cells (hASCs)
Source: Polymers (Basel). 2018 Sep 28;10(10):1073. doi: 10.3390/polym10101073 (PMC6403585; doi:10.3390/polym10101073)

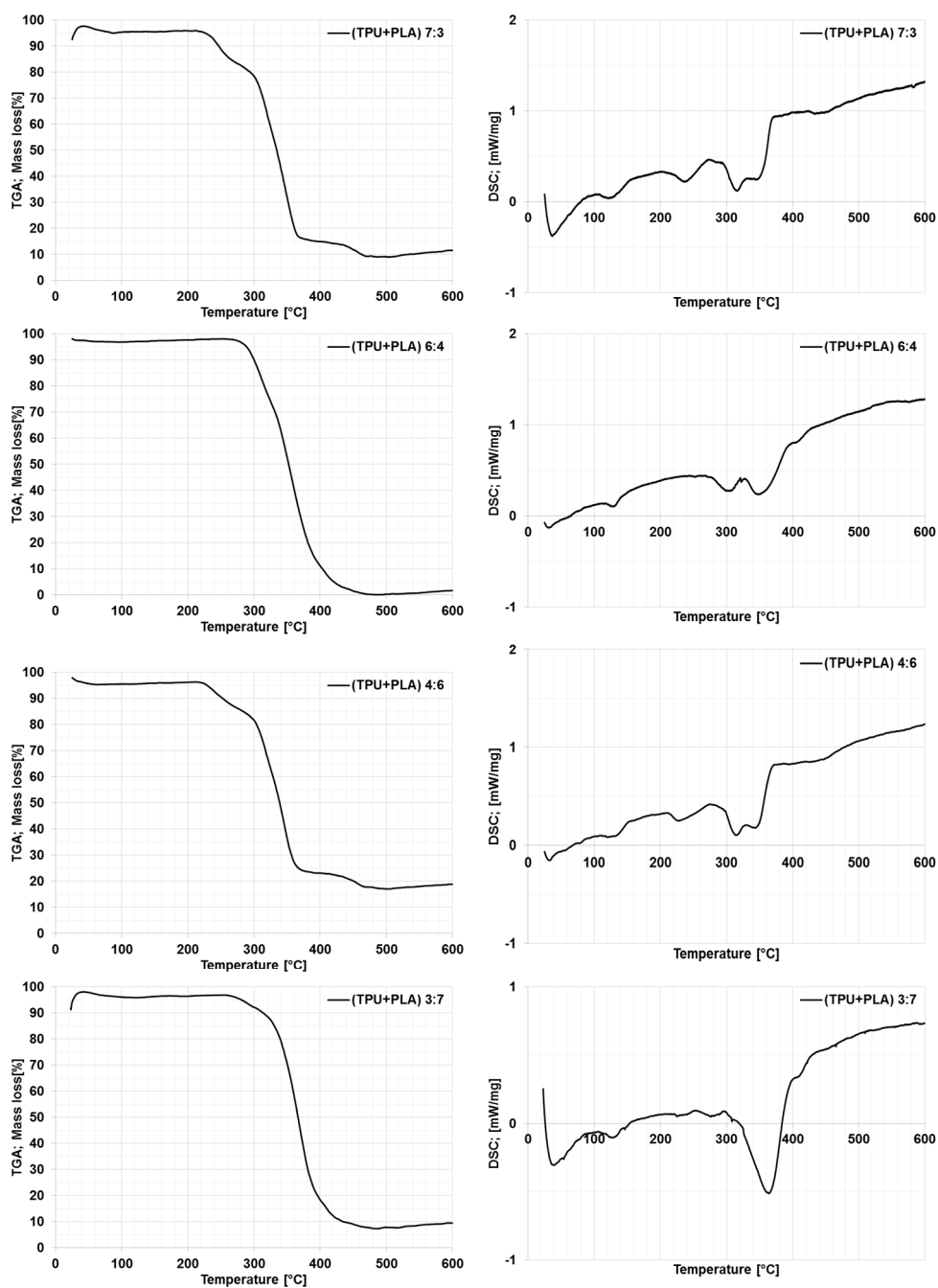

**Supplementary Figure S1.** TGA and DSC curves of different (TPU+PLA) blends: 7:3, 6:4, 4:6 and 3:7.

Supplement: Supplementary file 1 [file polymers-10-01073-s001.pdf]
